# The emerging role of the cerebellum in the affective theory of mind in the behavioral variant of frontotemporal dementia

**DOI:** 10.1007/s00415-024-12595-8

**Published:** 2024-08-06

**Authors:** Sonia Di Tella, Maria Caterina Silveri, Davide Quaranta, Naike Caraglia, Libera Siciliano, Camillo Marra, Maria Leggio, Giusy Olivito

**Affiliations:** 1 Department of Psychology, Università Cattolica del Sacro Cuore, Milan, Italy; 2 Department of Neuroscience, Università Cattolica del Sacro Cuore, Rome, Italy; 3 Neurology Unit, IRCCS Policlinico Universitario “A. Gemelli”, Rome, Italy; 4 Department of Psychology, Sapienza University of Rome, Via Dei Marsi 78, 00185 Rome, Italy; 5 Ataxia Research Laboratory, IRCCS Santa Lucia Foundation, Rome, Italy

## Dear Sirs,

In a previous study, Olivito et al. [6] showed significant grey matter (GM) loss in some cerebellar regions, mainly right and left crus I and left crus II, in patients with behavioral variant of frontotemporal dementia (bvFTD). In addition, functional connectivity (FC) changes were identified between these regions and supratentorial cortical areas critical for mentalization processes, supporting pivotal converging evidence on the role of the cerebellum in social cognition [12]. Interestingly, patterns of change in cerebello-cerebral FC correlated with performance on affective theory of mind (ToM) as assessed by the reading the mind with the eyes test (RMET, [1]). Earlier studies have reported that individuals with bvFTD perform poorly on a wide range of tests assessing ToM [2, 8], and suggested that ToM decline might be one of the best predictors for diagnosis [8].

In the present study, we aimed to extend our previous investigations on cerebello-cerebral FC changes and ToM disorders [6] to both components of ToM, cognitive and affective. To this end, we adopted the Yoni task ([9]; Italian validation by [5]). This test allows to explore the two components of ToM by matching affective and cognitive items in terms of perceptual load and level of complexity (first- and second-order inference) [9], limiting the influence of language, memory, and attentional factors on the performance.

In the present paper, we started from MRI data previously collected on a cohort of 15 bvFTD patients and 34 healthy subjects (HS) and published in Olivito and colleagues [6] while behavioral analyses were conducted on unpublished data previously collected from the administration of a computerized version of the Yoni task, as implemented on Qualtrics (https://www.qualtrics.com/), in the same cohort of 15 bvFTD patients (mean age/SD: 69.8/5.6 years; M/F = 11/4; mean educational level/SD: 11.1/4.8 years) (for further details on patients’ sample, see Olivito et al., [6]). For this analysis, a novel group of 16 age–sex–education-matched HS (mean age/SD: 68.2/6.1; M/F = 8/8; mean educational level/SD: 13.9/3.6) was recruited since Yoni task data were not available in the HS cohort used in Olivito and colleagues [6]. The task consists of 98 items, 84 mental (cognitive and affective), and 14 physical items (control items) at the first and second levels of inference to check general comprehension of the task instructions. The total ToM accuracy score (total ToM) is obtained by summing the scores of the first- and second-order items and dividing the sum by the total number of mental items (84) [(1st order ToM + 2nd order ToM)/84]. Four subtotals can also be derived: first-order ToM (range 0–24), second-order ToM (range 0–60), affective ToM (range 0–48), and cognitive ToM (range 0–36).

A generalized linear mixed-effects model (GLMM) was run to explore interactions between groups and within-task conditions, including ‘group’ (bvFTD, HS), ‘component’ (affective, cognitive, physical), ‘level’ (first, second order) and their interactions as fixed effects, ‘subjects’ as a random effect, and age and education as covariates. False discovery rate (FDR) correction was applied to adjust for multiple comparisons with the ‘*p.adjust’* package (https://rdrr.io/cran/POSTm/man/p.adjust.html*)* implemented in RStudio statistical software (version 2023.03.0), which given a set of *p* values, returns *p* values adjusted, setting the statistical threshold at *p* < 0.05.

**Fig. 1:**
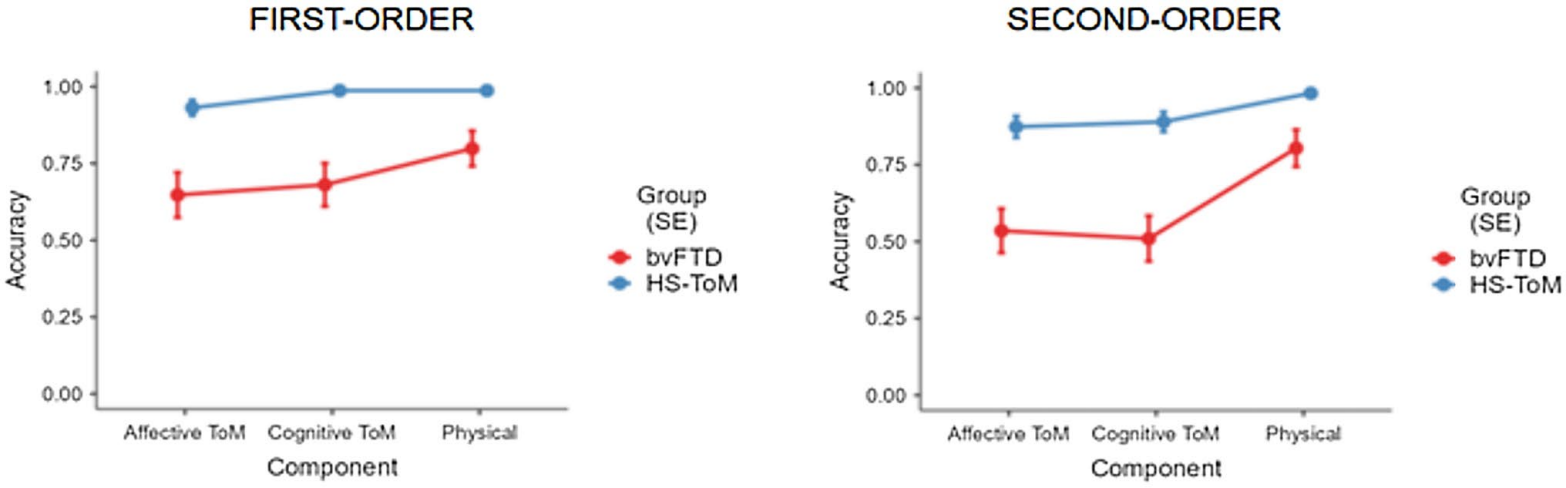
Graphical illustration of Yoni task performances obtained by bvFTD and HS-ToM groups. *ToM* theory of mind

The GLMM (*R*^2^ = 0.47) revealed a main effect of group (*χ*^2^ (1) = 27.54; *p* < 0.001) with the bvFTD performing worse than the HS. The interaction ‘group ✻ component’ (*χ*^2^ (2) = 7.85; *p* = 0.020) showed that patients performed worse on mental items (both cognitive and affective) compared to physical items (pFDR < 0.001), with no differences between ToM subcomponents (pFDR > 0.1). In contrast, HS showed comparable accuracy on mental ToM items and physical items (pFDR > 0.1). The significant ‘component ✻ level’ (*χ*^2^ (2) = 7.67; *p* = 0.022) interaction revealed lower accuracy for more complex second-order mental items (affective ToM–second vs. first order: pFDR = 0.003; cognitive ToM–second vs. first order: pFDR < 0.001), but not for physical control items (second vs. first order: pFDR > 0.1). See Fig. 1 for a visual representation of Yoni task performance in the bvFTD and HS-ToM groups.

Spearman’s correlation was calculated between the patients’ Yoni task scores (total, affective, cognitive, and physical items; items of first and second level of inference were collapsed) and increased FC involving bilateral cerebellar Crus I and key social brain regions as emerged by the seed-based FC analysis performed in our previous study (see Olivito et al., [6]).

The statistical threshold for correlation analysis was also set at *p* < 0.05. The results showed that Yoni total scores were negatively correlated with pairwise increased cerebello-cerebral FC between the left Crus I and left angular gyrus (*r* = − 0.660, *p* = 0.010) and the left Crus I and left precuneus (*r* = − 0.589, *p* = 0.027). Similarly, Yoni scores on affective items were negatively correlated with increased FC between left Crus I and left angular gyrus (*r* = − 0.756, *p* = 0.002) and left Crus I and left precuneus (*r* = − 0.699, *p* = 0.005). Cognitive item scores were negatively correlated with increased FC between right Crus I and left angular gyrus (*r* = − 0.634, *p* = 0.015) and right Crus I and left supramarginal gyrus (*r* = − 0.534, *p* = 0.049).

**Fig. 2:**
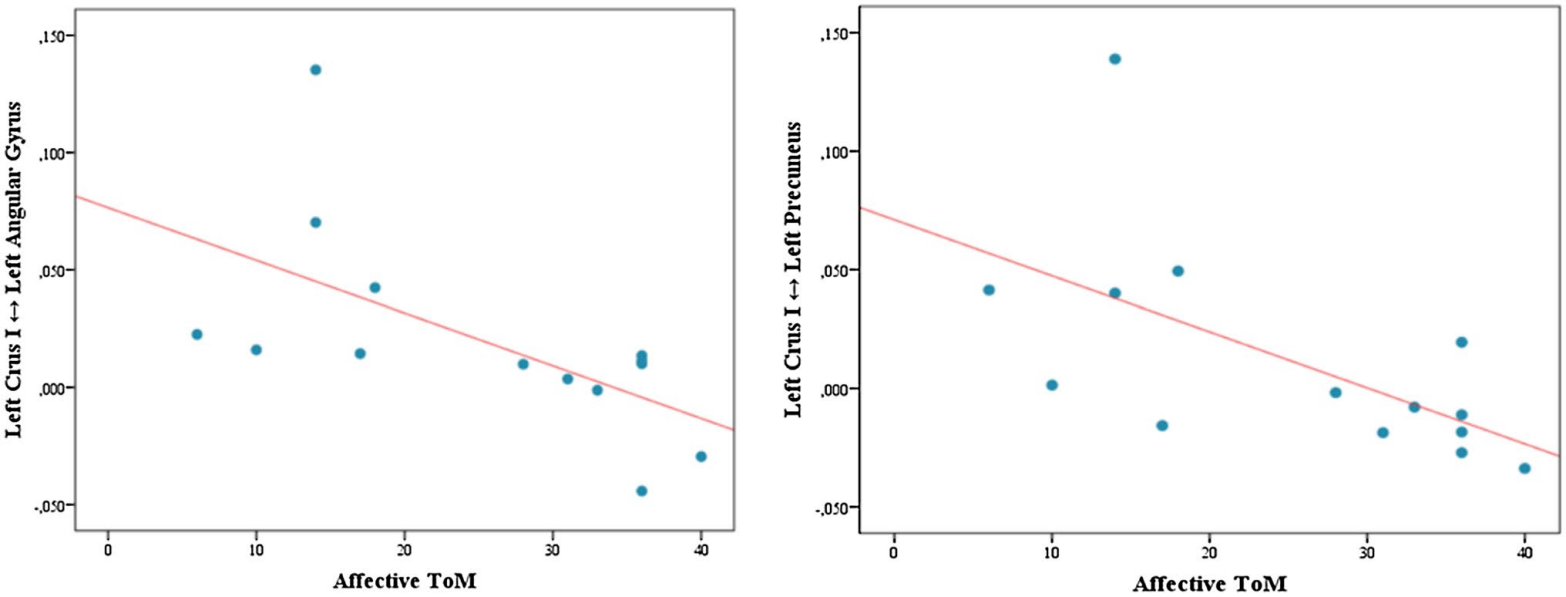
Scatterplots of correlations. Data scatterplots of significant correlations between Yoni affective accuracy scores and cerebello-cerebral overconnectivity patterns. Statistical significance at *p* < 0.05 after FDR correction for multiple comparisons. *ToM* theory of mind

No correlations were found between physical item scores and altered cerebello-cerebral FC (*p* < 0.1). It is worth noting that after applying FDR correction for multiple comparisons in the statistical software RStudio, only correlations between cerebello-cerebral FC and Yoni scores on affective items survived (left Crus I-angular gyrus: *p* = 0.012; left Crus I-left precuneus: *p* = 0.015) (Fig. 2).

The results of the current study confirm the validity of our previous observation on the role of the cerebellum in supporting the affective component of ToM in bvFTD [6]. Using the Yoni test, we were able to show that both the cognitive and affective ToM are altered in bvFTD, although data from the literature seem to indicate a more severe impairment of the affective component in bvFTD [7, 10]. Most importantly, our results provide additional insights into the putative role of the cerebello-cerebral mentalizing network, suggesting that FC alterations between core social cerebellar and cerebral regions [6] mostly involve the affective component of the ToM in bvFTD patients, regardless of the task used, the Yoni task (used here) or the RMET (as in the previous paper) [6]. The critical role of the cerebellum in social mentalizing has been widely demonstrated [12]. In particular, resting-state fMRI studies [11] have shown that the posterior cerebellar Crus I and II are functionally coupled to default mode regions specifically involved in social mentalizing, including the precuneus and angular gyrus [13]. In the present paper, we could confirm that functional overconnectivity between the cerebellar Crus I and mentalizing cerebral regions, specifically the precuneus and angular gyrus, correlates with the performance of bvFTD patients on tasks exploring the affective component of ToM, such as the affective items of the Yoni.

The present study provides the first evidence that the cerebellum, in concert with supratentorial regions, mainly contributes to the impairment of the affective component of ToM in patients with bvFTD. Indeed, while bvFTD patients showed impaired performances in both cognitive and affective mental items compared to controls, only affective Yoni scores were negatively correlated with overconnectivity between cerebello-cerebral pairwise regions, i.e., the Crus I and precuneus and angular gyrus, suggesting that low performance on affective items is related to altered cerebello-cerebral connectivity. Notably, while the angular gyrus and precuneus are known to be part of a shared neural network underlying affective and cognitive mentalizing [3], the present study emphasizes that, within this network, the cerebellum is mainly recruited during affective mentalizing in bvFTD. It has to be mentioned that, in spite of the stronger association between the affective component of ToM and cerebello-cerebral connectivity, the lack of statistical significant correlation between cognitive component of ToM and cerebello-cerebral connectivity after multiple comparison correction should be interpreted with caution and may also be due to low statistical power because of the small sample size.

Network-selective vulnerability of the cerebellum across different neurodegenerative disease has been previously described. In particular, it has been shown that cerebellar atrophied regions share robust and selective intrinsic connectivity with the atrophied regions in the cerebral cortex suggesting that distinct and circumscribed atrophy in the cerebellum reflects the disruption of specific cerebello-cerebral connections [4], thus subtending syndrome-specific profiles. In the context of social domain, this evidence suggests that the cognitive and affective processes subtended by the cerebellum could be selectively compromised in different age-related neurodegenerative conditions and that specific cerebellar circuits, which share extensive connections with the cerebral cortex, could be targeted by major neurodegenerative diseases [4]. In conclusion, as hypothesized by Olivito and colleagues [6], the pattern of cerebello-cerebral overconnectivity may reflect the pathological manifestation of an altered functional interaction within specific cerebello-cerebral circuits that might alter the affective ToM performances of patients with bvFTD.

## Data Availability

The data presented in this study are available on request from the corresponding author.
